# Correction: Piloting the informed health choices resources in Barcelona primary schools: A mixed methods study

**DOI:** 10.1371/journal.pone.0326589

**Published:** 2025-06-17

**Authors:** The PLOS One Staff

The author initials appear incorrectly in the citation. The correct citation is: Samsó Jofra L, Alonso-Coello P, Cánovas Martínez E, de Britos Marsal C, Gallego Iborra A, Niño de Guzman Quispe EP, et al. (2023) Piloting the informed health choices resources in Barcelona primary schools: A mixed methods study. PLOS ONE 18(7): e0288082. https://doi.org/10.1371/journal.pone.0288082.

The publisher apologizes for the error.
