# Supplementary material for: Piloting the informed health choices resources in Barcelona primary schools: A mixed methods study
Source: PLoS One. 2023 Jul 7;18(7):e0288082. doi: 10.1371/journal.pone.0288082 (PMC10328314; doi:10.1371/journal.pone.0288082)
Supplement: S1 File — (PDF) [file pone.0288082.s001.pdf]

Piloting the Informed Health Choices resources in Barcelona primary schools: A mixed methods study

Supporting information

S1 File. Study variables

	Quantitative variable		Qualitative variable
	Categorical variable	Continuous variable	
**Quantitative data collection**			
**Intervention in the schools**			
Participants; IHC-Barcelona Working Group	X		
Participants; Students	X		
Participants; Class	X		
Participants; Teachers	X		
Participants; Informed consent from students' families	X		
Participants; Workshop	X		
Workshop		X	
Lessons		X	
CLAIM test - Response rate	X		
CLAIM test - Passing score	X		
CLAIM test - Mastery score	X		
**Assessment of the IHC resources by the teachers before the lessons (questionnaire)**			
Students' understandability, desirability, suitability, and usefulness		X	
Teachers' understandability, desirability, suitability, and usefulness		X	
Examples of treatment claims			X [a]
**Assessment of the lessons by the teachers after a lesson (questionnaire)**			
Students' understandability, desirability, suitability, and usefulness		X	
Teachers' understandability, desirability, suitability, and usefulness		X	
Technique used to teach the lessons	X		X [a]
Facilitators and barriers to teach the lessons	X		
Suggestions to improve the lessons			X [a]
**Overall assessment of the IHC resources by the teachers at the end of the lessons (questionnaire)**			
Students' overall understandability, desirability, suitability, and usefulness		X	
Teachers' overall understandability, desirability, suitability, and usefulness		X	
Suggestions to improve the IHC resources			X [a]
**Qualitative data collection**			
**Non-participatory observations during the lessons**			
Students' understandability, desirability, suitability, and usefulness			X
Teachers' understandability, desirability, suitability, and usefulness			X
Technique used to teach the lessons			X
Facilitators and barriers to teach the lessons			X
Examples of treatment claims			X

**Semi-structured interviews with the students after a lesson**	
Students' understandability, desirability, suitability, and usefulness	X
Examples of treatment claims	X
Suggestions to improve the lessons	X

Abbreviations: IHC: Informed Health Choices.

[a] Open-ended questions
