# Supplementary material for: Piloting the informed health choices resources in Barcelona primary schools: A mixed methods study
Source: PLoS One. 2023 Jul 7;18(7):e0288082. doi: 10.1371/journal.pone.0288082 (PMC10328314; doi:10.1371/journal.pone.0288082)
Supplement: S2 File — (PDF) [file pone.0288082.s002.pdf]

Piloting the Informed Health Choices resources in Barcelona primary schools: A mixed methods study

Supporting information

S2 File. Good Reporting of A Mixed Methods Study (GRAMMS) checklist [1]

	Item	Assessment
1	Describe the **justification** for using a mixed methods approach to the research question.	We reported the **objectives and justification** of methods in the Background section:<br><br>"Currently, there are no specific learning resources to teach primary school children to think critically about their health in Spanish context. The IHC-Barcelona Working Group translated the IHC resources into Spanish [27-29]. The next step was to pilot test these resources and ensure their appropriateness for Spanish primary school children. For this purpose, we conducted this mixed methods study aimed to 1) explore the students' and teachers' experience when using the IHC primary school resources in Barcelona (Spain), and 2) formulate recommendations to use the IHC primary school resources in this setting."
2	Describe the design in terms of the **purpose, priority, and sequence** of methods.	We reported **purpose, priority, and sequence** in the Methods section:<br><br>"We conducted a convergent mixed methods study [30]. We used multiple approaches to collect in parallel quantitative data (teachers' questionnaires) and qualitative data (lessons' non-participatory observations [NPOs], and students' semi-structured interviews [SSIs]) on users' experience with the IHC resources (S1 File). The quantitative and qualitative data were collected, analysed, and interpreted separately. Finally, we integrated the qualitative and quantitative findings for an in-depth understanding of the same phenomenon (triangulation purpose) [31]."
3	Describe each method in terms of **sampling, data collection and analysis**.	We reported **sampling, data collection and analysis** in the Methods section:<br><br>**"Participants**<br>Quantitative sample<br>All the teachers who participated in the intervention completed ad hoc self-administered questionnaires (before the lessons, after each lesson, and at the end of the lessons) to explore the users' experience with the IHC resources.<br><br>Qualitative sample<br>We selected a convenience sample of lessons to conduct non-participatory observations (NPOs) during the lessons and semi-structured interviews (SSIs) with students after the lessons. We expected to perform at least two NPOs from each lesson in randomly assigned classes (18 observations) and two SSIs of each lesson on randomly assigned students (18 interviews).<br><br>**Data collection**<br>Quantitative data<br>• Assessment of the IHC resources by the teachers before the lessons<br>• Assessment of the lessons by the teachers after a lesson<br>• Overall assessment of the IHC resources by the teachers at the end of the lessons<br>Qualitative data<br>• Non-participatory observations during the lessons<br>• Semi-structured interviews with the students after a lesson<br><br>**Data analysis**<br>Quantitative analysis<br>We conducted a descriptive analysis of the categorical variables (absolute and relative frequencies), and the continuous variables (median and range) (S1 File). We only considered the CLAIM test results for the students at schools that completed all the lessons. We calculated the proportion of students with a passing score (basic knowledge of the concepts and how to apply them, 13 points or more over 24), and the proportion of the students with a mastery score (clear knowledge of the concepts and how to apply them, 20 points or more over 24) [39].<br><br>Qualitative analysis<br>We analysed qualitative data derived from lessons' NPOs, students' SSIs, and free-text responses of teachers' questionnaires (S1 File). We conducted a framework deductive analysis [40] for qualitative data related to several domains of an adapted version of a user-experience honeycomb framework: understandability, desirability, suitability, and usefulness [38]. We applied the following steps: 1) categorisation of quotes using the framework's themes, and 2) proposal of

		subthemes under the themes. We conducted a thematic inductive analysis for qualitative data not suitable for the framework: technique used to teach the lessons, facilitators and barriers to teach the lessons, examples of claims about treatment effects, and suggestions to improve the lessons. We applied the following steps: 1) codification of quotes, 2) proposal of descriptive themes, and 3) identification of analytic themes. One researcher categorised and coded quotes, and proposed themes and subthemes independently. A second researcher cross-checked codes, corresponding quotes, themes, and subthemes. Disagreements were initially solved by consensus; if necessary, a third reviewer was consulted."
4	Describe where **integration** has occurred, **how** it has occurred and **who** has participated in it.	We reported **integration analysis** in the Methods section:<br><br>"We merged quantitative and qualitative results using a joint display to compare and validate the findings [41]. We applied the following steps: 1) mapping quantitative and qualitative results by outcome into a summarized matrix, 2) exploring the convergence (findings from quantitative and qualitative approach agree), complementarity (findings from each approach offer complementary information), or discrepancy (findings from each approach appear to be contradictive) [42], and 3) narrative synthesising of the integration findings. One reviewer conducted the integration analysis independently. A second reviewer cross-checked the summarised results matrix and the narrative synthesis. Disagreements were initially solved by consensus; if necessary, a third reviewer was consulted."
5	Describe any **limitation** of one method associated with the present of the other method.	We reported **limitations** in the Discussion section:<br><br>"Finally, it was a challenge to use a mixed method approach since it requires expertise in quantitative and qualitative methods, and in the combination of both."
6	Describe any **insights** gained from mixing or integrating methods.	We reported **strengths** in the Discussion section:<br><br>"Finally, we used a mixed methods approach, with quantitative and qualitative data collection and integration analysis, to obtain comprehensive and validated findings and draw more rigorous and reliable recommendations."

Reference: O'Cathain A, Murphy E, Nicholl J. The quality of mixed methods studies in health services research. J Health Serv Res Policy. 2008;13(2):92-8.
