# Supplementary material for: Piloting the informed health choices resources in Barcelona primary schools: A mixed methods study
Source: PLoS One. 2023 Jul 7;18(7):e0288082. doi: 10.1371/journal.pone.0288082 (PMC10328314; doi:10.1371/journal.pone.0288082)
Supplement: S3 File — (PDF) [file pone.0288082.s003.pdf]

Piloting the Informed Health Choices resources in Barcelona primary schools: A mixed methods study

Supporting information

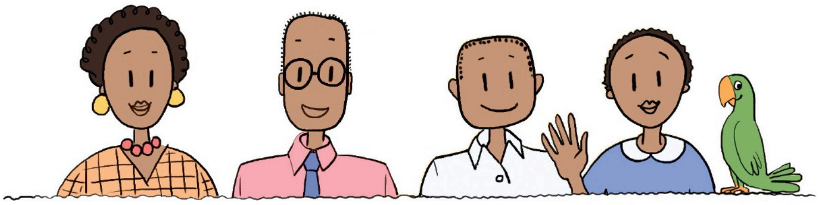

S3 File. Workshop programme

Date: November 15, 2019; 9:00-14:30h
Place: Seminar 2, Faculty of Medicine UAB, Hospital de la Santa Creu i Sant Pau, Barcelona

	**Welcome and introductions**
**9:00 – 9:30h**	**Opening session "What is Evidence-Based Medicine?"**
**9:30 – 10:00h**	**Presentation of the Informed Health Choices project**<br>• What is the project about and why is it important?<br>• Who has created these resources and how have they been created?<br>• What are the resources?<br>• Questions, comments, or suggestions
**10:00 – 11:00h**	**Presentation of the study “Piloting the Informed Health Choices resources in Barcelona primary schools: A mixed methods study”**<br>• Introduction of the study objectives<br>• Introduction of the study methods<br>• Introduction of the study work plan<br>• Questions, comments, or suggestions
**11:00 – 11:30h**	**Coffee break and group photo**
**11:30 – 12:30h**	**Roleplay session “How do we teach a lesson? We practice with Lesson 7 - Fair Comparisons with Many People"**<br>• Introduction of the lesson's key concept<br>• Read-aloud of the lesson, completion of the activity and the exercises<br>• Questions, comments, or suggestions
**12:30 – 13:00h**	**Introduction of the plan to teach the lessons in School 1**
**13:00 – 13:30h**	**Introduction of the plan to teach the lessons in School 2**
**13:30 – 14:00h**	**Introduction of the plan to teach the lessons in School 3**
**14:00 – 14:30h**	**Closing remarks**

Iberoamerican Cochrane Centre - Sant Pau Biomedical Research Institute
Hospital de la Santa Creu i Sant Pau. Building 18, ground floor.
C/ Sant Antoni Maria Claret 167. 08025 Barcelona, Spain. Tel. (+34) 93 553 78 14
Contact: Laura Martínez García, laura.martinez.garcia@cochrane.es
