# Supplementary material for: Piloting the informed health choices resources in Barcelona primary schools: A mixed methods study
Source: PLoS One. 2023 Jul 7;18(7):e0288082. doi: 10.1371/journal.pone.0288082 (PMC10328314; doi:10.1371/journal.pone.0288082)
Supplement: S4 File — (PDF) [file pone.0288082.s004.pdf]

# Piloting the Informed Health Choices resources in Barcelona primary schools: A mixed methods study

# Supporting information

## S4 File. List of the key concepts included in the Informed Health Choices learning resources for primary school children [1]

| n | Concepts (short titles) | Explanations |
|---|---|---|
| **Claims**<br>Claims about effects that are not supported by evidence from fair comparisons are not necessarily wrong, but there is an insufficient basis for believing them. | | |
| 1 | **Do not assume that treatments are safe ("100% safe!")** | People often exaggerate the benefits of treatments and ignore or downplay potential harms. However, few effective treatments are 100% safe. |
| 2 | **Do not assume that comparisons are not needed ("No comparison needed!")** | Unless a treatment is compared to something else, it is not possible to know what would happen without the treatment. |
| 3 | **Do not assume that a single study is a sufficient basis for a claim about treatment effects ("A study shows!")** | The results of one study considered in isolation can be misleading. A single comparison of treatments rarely provides conclusive evidence; and results are often available from other comparisons of the same treatments. |
| 4 | **Do not assume that a treatment is helpful or safe based on how widely used it is or has been ("Old is better!")** | Treatments that have not been properly evaluated but are widely used or have been used for a long time are often assumed to work. Sometimes, however, they may be unsafe or of doubtful benefit. |
| 5 | **Do not assume that a treatment is better based on how new or technologically impressive it is ("New is better!")** | New treatments can be assumed to be better simply because they are new, more expensive, or technologically impressive. However, on average, they are only very slightly likely to be better than other available treatments. Some side effects of treatments, for example, take time to appear, and without long term follow-up it may not be possible to know whether they will appear. |
| 6 | **Do not assume that there are no competing interests ("As advertised!")** | People with an interest in promoting a treatment (in addition to wanting to help people) – for example, to make money – may promote treatments by exaggerating benefits, ignoring potential harmful effects, cherry picking which information is used, or making false claims. Conversely, people may be opposed to a treatment for a range of reasons, such as cultural practices. |
| 7 | **Do not assume that personal experiences alone are sufficient ("It worked for me!")** | People can be led to believe that improvements in a health problem (for example, recovery from a disease) resulted from having received a treatment. Similarly, they might believe that an undesirable health outcome was due to having received a treatment. However, the fact that an individual recovered after receiving a treatment does not mean that the treatment caused the improvement, or that other people receiving the same treatment will also improve. The improvement (or the undesirable health outcome) might have occurred even without treatment. |
| 8 | **Opinions alone are sufficient ("Recommended by experts!")** | People often disagree about the effects of treatments, including doctors, researchers, and patients. This may be because their opinions are not always based on systematic reviews of fair comparisons of treatments. Who makes a treatment claim, how likable they are, or how much experience and expertise they have do not provide a reliable basis for assessing how reliable their claim is. This does not mean that conflicting opinions should be given equal weight – or that the existence of conflicting opinions means that no conclusion can be reached. How much weight to give an opinion should be based on the strength of the evidence supporting it. |
| **Comparisons**<br>To identify treatment effects, studies should make fair comparisons, designed to minimise the risk of systematic errors (biases) and random errors (the play of chance). | | |
| 9 | **Consider whether the people being compared were similar (Dissimilar comparison groups)** | If people in treatment comparison groups differ in ways other than the treatments being compared, the apparent effects of the treatments might reflect those differences rather than actual treatment effects. Differences in the characteristics of |

| | | |
|---|---|---|
| | | the people in the comparison groups at the beginning of the comparison might result in estimates of treatment effects that appear either larger or smaller than they actually are. A method such as allocating people to different treatments by assigning them random numbers (the equivalent of flipping a coin) is the best way to ensure that the groups being compared are similar in terms of both measured and unmeasured characteristics. |
| 10 | **Consider whether the people being compared knew which treatments they received**<br>**(Dissimilar expectations or behaviours)** | People in a treatment group may behave differently or experience improvements or deterioration because they know the treatment to which they have been assigned. If this phenomenon is associated with an improvement in their symptoms it is known as a placebo effect; if it is associated with a harmful effect it is known as a nocebo effect. If individuals know that they are receiving a treatment that they believe is either better or worse than an alternative (that is, they are not "blinded"), some or all the apparent effects of treatments may be due either to placebo or nocebo effects. |
| 11 | **Be cautious of small studies**<br>**(Few people or events)** | When there are few outcome events, differences in outcome frequencies between the treatment comparison groups may easily have occurred by chance and may mistakenly be attributed to differences in the effects of the treatments, or the lack of a difference. |
| **Choices**<br>What to do depends on judgements about a problem, the relevance of the available evidence, and the balance of expected benefits, harms, and costs. | | |
| 12 | **Consider how important each advantage and disadvantage is when weighing the pros and cons and making choices**<br>**(Do the advantages outweigh the disadvantages for you?)** | Estimates of benefits and harms depend on how much weight people give to treatment advantages and disadvantages. Different people may value outcomes differently and sometimes make different choices because of this. In addition, people usually place more value on outcomes that happen soon than on outcomes that happen years into the future. In other words, the further into the future an outcome (for example, reducing the chance of heart disease or cancer after many years) the more people tend to "discount" its value or importance. The balance between the advantages and disadvantages of treatments may also depend on how much costs and events in the future are discounted. |

Reference: Oxman AD, Chalmers I, Dahlgren A, and the Informed Health Choices Group. Key Concepts for assessing claims about treatment effects and making well-informed treatment choices (Version 2022). IHC Working Paper. 2022.
