# Supplementary material for: Piloting the informed health choices resources in Barcelona primary schools: A mixed methods study
Source: PLoS One. 2023 Jul 7;18(7):e0288082. doi: 10.1371/journal.pone.0288082 (PMC10328314; doi:10.1371/journal.pone.0288082)
Supplement: S5 File — (PDF) [file pone.0288082.s005.pdf]

Piloting the Informed Health Choices resources in Barcelona primary schools: A mixed methods study

Supporting information

S5 File. *Ad hoc* questionnaires and guides

S5.1 File. Questionnaire for the assessment of the IHC resources by the teachers before the lessons 2
S5.2 File. Questionnaire for the assessment of the lessons by the teachers after a lesson 4
S5.3 File. Questionnaire for the overall assessment of the IHC resources by the teachers at the end of the lessons 9
S5.4 File. Guide for the non-participatory observations during the lessons 11
S5.5 File. Guide for the semi-structured interviews with the students after a lesson 16

S5.1 File. Questionnaire for the assessment of the IHC resources by the teachers before the lessons

1. Objective

- Explore the teachers' initial perception of the IHC resources.

2. Questionnaire identification

1	**Date** (DD/MM/YYYY)	
2	**Teacher** (surname, name)	
3	**School**	
4	**Class**	

Assess the aspects below using the following scale: completely disagree, disagree, neither agree or disagree, agree, and completely agree. Mark your answer with an "X".

	Completely disagree ☹	Disagree	Neither agree or disagree 😑	Agree	Completely agree ☺

3. Assessment of the students' expected experience with the IHC resources

5	**I expect the students will UNDERSTAND the content of the resources.**					
6	**I expect the students will show INTEREST in the content of the resources (listening, focused attention, and active participation).**					
7	**I expect the students will be able to APPLY the content of the resources to their daily life (e.g., claim search in their context).**					
8	**I expect the IHC resources will be USEFUL for students to learn to think critically about health.**					

4. Assessment of the teachers' experience with the IHC resources

9	**I have UNDERSTOOD the content of the resources.**					
10	**I have been INTERESTED in the content of the resources.**					
11	**I have identified situations in my daily live where I can APPLY the content of the resources (e.g., claim search in my context).**					

**12**	**<u>The Health Choices Book</u> appears to be USEFUL to teach to think critically about health.**					
**13**	**The <u>Teachers' Guide</u> appears to be USEFUL to teach to think critically about health.**					
**14**	**The <u>Activity Cards</u> appear to be USEFUL to teach to think critically about health.**					
**15**	**The <u>Checklist Poster</u> appears to be USEFUL to teach to think critically about health.**					

5. Example of a TREATMENT CLAIM

**16**	**Can you remember any TREATMENT CLAIM that you may have heard in your context?**	☐ Yes ☐ No ☐ Don't know/No response

Please, describe the example.

**17**	**Claim**<br>(e.g. cow dung heals burns!)	
**18**	**Treatment**<br>(e.g. putting cow dung on a burn)	
**19**	**Effect**<br>(e.g. healing burns)	
**20**	**Basis**<br>(e.g. someone's personal experience)	
**21**	**Is the claim reliable?**<br>(e.g. no)	☐ Yes ☐ No ☐ Not sure

S5.2 File. Questionnaire for the assessment of the lessons by the teachers after a lesson

1. Objectives

- Assess (self-reportedly) the degree of implementation of the IHC resources.
- Explore the teachers' experience when using the IHC resources.

2. Questionnaire identification

1	**Date** (DD/MM/YYYY)	
2	**Teacher** (surname, name)	
3	**School**	
4	**Class**	
5	**Number of students**	
6	**Lesson number**	
7	**Lesson start time**	
8	**Lesson finish time**	

Assess the aspects below using the following scale: completely disagree, disagree, neither agree or disagree, agree, and completely agree. Mark your answer with an "X".

	**Completely disagree** ☹	**Disagree**	**Neither agree or disagree** 😑	**Agree**	**Completely agree** ☺

3. Assessment of the students' experience with the IHC resources

9	**The students appear to have UNDERSTOOD the content of the lesson.**					
10	**The students appear to show INTEREST in the content of the lesson (listening, focused attention, and active participation).**					
11	**The students appear to be able to APPLY the content of the lesson to their daily life (e.g., claim search in their context).**					
12	**<u>The Health Choices Book</u> was USEFUL for students to learn this lesson.**					
13	**The <u>Activity Cards</u> were USEFUL for students to learn this lesson (if applicable).**					
14	**The <u>Checklist Poster</u> was USEFUL for students to learn this lesson.**					

4. Assessment of the teachers' experience with the IHC resources

15	**I have UNDERSTOOD the content of the lesson.**					
16	**I have been INTERESTED in the content of the lesson.**					
17	**I have identified situations in my daily live where I can APPLY the content of the lesson (e.g., claim search in my context).**					
18	**The Health Choices Book was USEFUL to teach this lesson.**					
19	**The Teachers' Guide was USEFUL to teach this lesson.**					
20	**The Activity Cards were USEFUL to teach this lesson (if applicable).**					
21	**The Checklist Poster was USEFUL to teach this lesson.**					

5. Assessment of the technique used to teach the lesson

22	**Has the last lesson been reviewed?** (step 1)	☐ Yes ☐ No ☐ Don't know/No response ☐ Not assessable
23	**Has the story (comic) been read?** (step 2)	☐ Yes ☐ No ☐ Don't know/No response ☐ Not assessable
24	**Has there been a discussion?** (step 3)	☐ Yes ☐ No ☐ Don't know/No response ☐ Not assessable
25	**Has the activity been completed?** (step 4)	☐ Yes ☐ No ☐ Don't know/No response ☐ Not assessable
26	**Have any of the exercises been completed?** (step 5)	☐ Yes ☐ No ☐ Don't know/No response ☐ Not assessable
27	**Have you used any other method and/or strategy to teach the lesson?**	☐ Yes ☐ No ☐ Don't know/No response
28	**Please, describe it/them here:**	

6. Assessment of the facilitators and barriers to teach the lesson

What do you think the main factors that have FACILITATED the use of the resources during the lesson have been?

**29**	**Teachers**	☐ Profiles and competences<br>☐ Understanding of the content being taught<br>☐ Sufficient training<br>☐ Self-efficacy<br>☐ Fit to the teacher's teaching style and context (e.g., class size)<br>☐ Attitudes<br>☐ Beliefs<br>☐ Emotions<br>☐ Motivation<br>☐ Positive learning environment
**30**	**Students**	☐ Literacy<br>☐ Attendance<br>☐ Motivation to learn<br>☐ Attitudes<br>☐ Beliefs<br>☐ Home environment<br>☐ Differentiated instruction<br>☐ Peer influence
**31**	**Learning resources**	☐ Value of the material<br>☐ Compatibility with the curriculum<br>☐ Appropriateness of the material<br>☐ Credibility of the material
**32**	**School system and environment**	☐ Time constraints<br>☐ Competing priorities<br>☐ School organisation and management<br>☐ School resources, particularly human resources<br>☐ Attitudes and beliefs of head teacher and other teachers<br>☐ Parent and community involvement<br>☐ Regulations<br>☐ Political environment<br>☐ Bureaucracy<br>☐ Incentives and disincentives
**33**	**Others**	

What do you think the main barriers that have HINDERED the use of the resources during the lesson have been?

**34**	**Teachers**	☐ Profiles and competences<br>☐ Understanding of the content being taught<br>☐ Sufficient training<br>☐ Self-efficacy<br>☐ Fit to the teacher's teaching style and context (e.g., class size)<br>☐ Attitudes<br>☐ Beliefs<br>☐ Emotions<br>☐ Motivation<br>☐ Positive learning environment

35	**Students**	☐ Literacy<br>☐ Attendance<br>☐ Motivation to learn<br>☐ Attitudes<br>☐ Beliefs<br>☐ Home environment<br>☐ Differentiated instruction<br>☐ Peer influence
36	**Learning resources**	☐ Value of the material<br>☐ Compatibility with the curriculum<br>☐ Appropriateness of the material<br>☐ Credibility of the material
37	**School system and environment**	☐ Time constraints<br>☐ Competing priorities<br>☐ School organisation and management<br>☐ School resources, particularly human resources<br>☐ Attitudes and beliefs of head teacher and other teachers<br>☐ Parent and community involvement<br>☐ Regulations<br>☐ Political environment<br>☐ Bureaucracy<br>☐ Incentives and disincentives
38	**Others**	

7. Suggestions to improve the lesson

39	**Would you change anything from this lesson?**	☐ Yes ☐ No ☐ Don't know/No response
40	**What would you change?**	
41	**Why would you change it?**	
42	**How would you change it?**	

8. Table of potential facilitators and barriers

**Teachers**		
**1**	Profiles and competences	Teacher’s education and experience in relation to the lessons being taught
**2**	Understanding of the content being taught	Teachers’ understanding of the context
**3**	Sufficient training	The extent to which the teachers received sufficient training in teaching the lessons
**4**	Self-efficacy	Teacher’s confidence in teaching the lessons
**5**	Fit to the teacher’s teaching style and context (e.g., class size)	Teachers’ comfort or ability to adapt the instructions to their style and context
**6**	Attitudes	Teachers’ attitude towards new resources (change), science, critical thinking and independent thinking by the student body (or their role as authorities in the classroom)
**7**	Beliefs	Teachers’ beliefs about the methods or content (e.g., what treatments work or the concepts)
**8**	Emotions	Teachers’ emotions, such as stress or anxiety
**9**	Motivation	Teachers’ motivation to teach the material
**10**	Positive learning environment	Teachers’ ability to create a positive learning environment; for example, encourage discussion, respond positively to questions, engage students
**Students**		
**11**	Literacy	Students’ ability to read and understand the material
**12**	Attendance	Students’ attendance or reasons for poor attendance (e.g., long distance to school or inability to pay school fees)
**13**	Motivation to learn	Students’ motivation to learn the new material
**14**	Attitudes	Students’ attitudes towards learning, towards authorities, towards science, towards critical thinking
**15**	Beliefs	Students’ beliefs about the content (e.g., what treatments work or the concepts)
**16**	Home environment	The extent to which the student’s home environment encourages or discourages learning from the lessons
**17**	Differentiated instruction	The extent to which students different learning needs are met
**18**	Peer influence	Positive or negative attitudes of other students towards the material
**Learning resources**		
**19**	Value of the material	The extent to which the materials are valued by the teachers and students
**20**	Compatibility with the curriculum	The extent to which the resources fits with the rest of the curriculum and how it is taught
**21**	Appropriateness of the material	The extent to which the resources are relevant, challenging and engaging
**22**	Credibility of the material	The extent to which the teachers and students perceive the resources as credible
**School system and environment**		
**23**	Time constraints	The extent to which there is sufficient time to accommodate introducing the new material
**24**	Competing priorities	The extent to which other priorities for the school, teachers or students limit introducing the resources (e.g., preparing for exams)
**25**	School organisation and management	The extent to which the school provides an environment that supports adoption of new subjects, resources and teaching methods
**26**	School resources, particularly human resources	The extent to which the school has adequate resources to introduce the new resources (e.g., human resources, student/teacher ratio, teacher workload, classroom space and classroom resources, such as blackboards and acoustics)
**27**	Attitudes and beliefs of head teacher and other teachers	Attitudes or beliefs of colleagues that influence the teacher’s interest in and ability to teach the material
**28**	Parent and community involvement	Parents’ attitudes towards the new resources or how things are done at the school
**29**	Regulations	Regulations (e.g., Ministry of Education policies and regulations) that affect introducing the new material
**30**	Political environment	Elements of the political environment that affect introducing the new material; for example, authoritarianism or teacher strikes
**31**	Bureaucracy	Bureaucratic arrangements that delay or limit introduction of the new materials, or facilitate introducing them
**32**	Incentives and disincentives	Incentives or disincentives to introduce the new resources for teachers or head teachers

S5.3 File. Questionnaire for the overall assessment of the IHC resources by the teachers at the end of the lessons

1. Objective

- Explore the teachers' final experience with IHC resources.
- Compare the initial perception and final experiences after using IHC resources.

2. Questionnaire identification

1	**Date** (DD/MM/YYYY)	
2	**Teacher**<br>(surname, name)	
3	**School**	
4	**Class**	

Assess the aspects below using the following scale: completely disagree, disagree, neither agree or disagree, agree, and completely agree. Mark your answer with an "X".

	Completely disagree ☹	Disagree	Neither agree or disagree 😑	Agree	Completely agree ☺

3. Assessment of the students' experience with IHC resources

5	**The students appear to have UNDERSTOOD the content of the resources.**					
6	**The students appear to show INTEREST in the content of the resources (listening, focused attention, and active participation).**					
7	**The students appear to be able to APPLY the content of the resources to their daily life (e.g., claim search in their context).**					
8	**The resources appear to be USEFUL for students to learn to think critically about health.**					

4. Assessment of the teachers' experience with the IHC resources

9	**I have UNDERSTOOD the content of the resources.**					
10	**I have been INTERESTED in the content of the resources.**					
11	**I have identified situations in my daily live where I can APPLY the content of the resources (e.g., claim search in my context).**					

**12**	**<u>The Health Choices Book</u> was USEFUL to teach to think critically about health.**					
**13**	**The <u>Teachers' Guide</u> was USEFUL to teach to think critically about health.**					
**14**	**The <u>Activity Cards</u> were USEFUL to teach to think critically about health (when applicable).**					
**15**	**The <u>Checklist Poster</u> was USEFUL to teach to think critically about health.**					

5. Suggestions to improve the IHC resources

**16**	**Would you change anything from the resources?**	☐ Yes ☐ No ☐ Don't know/No response
**17**	**What would you change?**	
**18**	**Why would you change it?**	
**19**	**How would you change it?**	

S5.4 File. Guide for the non-participatory observations during the lessons

1. Objectives

- Assess (objectively) the degree of implementation of the IHC resources.
- Explore the students' and teachers' experience when using the IHC resources.

2. Instructions

1. Live observation of the lesson.
2. Do not participate during the lesson and try to go unnoticed.
3. Follow the lesson using the Teachers' Guide.
4. Fill out the questionnaire during the observation.
5. Briefly explain to the teacher what the non-participatory observations is about:
    - "We are not testing you."
    - "We are testing the IHC resources (what parts of the lessons work well and less well in the class context)."
    - "You should carry out the lessons the same way that they would if you were not being observed (as much as that is possible)."

3. Questionnaire identification

1	**Date** (DD/MM/YYYY)	
2	**Researcher** (surname and name)	
3	**Teacher** (surname, name)	
4	**School**	
5	**Class**	
6	**Number of students**	
7	**Lesson number**	
8	**Lesson start time**	
9	**Lesson finish time**	
10	**Recording**	☐ Yes ☐ No ☐ Don't know/No response

4. Assessment of the students' experience with the IHC resources

11	**The students appear to have UNDERSTOOD the content of the lesson.**	
12	**The students appear to BE INTERESTED in the content of the lesson (listening, focused attention and active participation).**	
13	**The students have identified situations in their daily lives where they can APPLY the content of the lesson (e.g., claim search in their context).**	
14	**<u>The Health Choices Book</u> was USEFUL for students to learn this lesson.**	

15	**The Activity Cards were USEFUL for students to learn this lesson (if applicable).**	
16	**The Checklist Poster was USEFUL for students to learn this lesson.**	

5. Assessment of the teachers' experience with the IHC resources

17	**The teacher appears to have UNDERSTOOD the content of the lesson.**	
18	**The teacher appears to BE INTERESTED in the content of the lesson.**	
19	**The teacher has identified situations in their daily live where they can APPLY the content of the lesson (e.g., claim search in their context).**	
20	**The Health Choices Book was USEFUL for teachers to teach this lesson.**	
21	**The Teachers' Guide was USEFUL for teachers to teach this lesson.**	
22	**The Activity Cards were USEFUL for teachers to teach this lesson (if applicable).**	
23	**The Checklist Poster was USEFUL for teachers to teach this lesson.**	

6. Assessment of the technique used to teach the lesson

24	**Has the last lesson been reviewed?** (step 1)	
25	**Has the story (comic) been read?** (step 2)	
26	**Has there been a discussion?** (step 3)	
27	**Has the activity been completed?** (step 4)	
28	**Have any of the exercises been completed?** (step 5)	
29	**Has the teacher used any other method and/or strategy to teach the lesson?**	

30	**Please, describe it/them here:**	

7. Assessment of the facilitators and barriers to teach the lesson

What do you think the main factors that have FACILITATED the use of the resources during the lesson have been?

(The table on potential facilitators and barriers is included at the end of the questionnaire).

31	**Teachers**	
32	**Students**	
33	**Learning resources**	
34	**School system and environment**	
35	**Others**	

What do you think the main barriers that have HINDERED the use of the resources during the lesson have been?

(The table on potential facilitators and barriers is included at the end of the questionnaire).

36	**Teachers**	
37	**Students**	
38	**Learning resources**	
39	**School system and environment**	

**40**	**Others**	

8. Example of a TREATMENT CLAIM

**41**	**Have the students/teacher described any TREATMENT CLAIM in their context during the lesson?**	☐ Yes ☐ No ☐ Don't know/No response

Please, describe the example.

**42**	**Claim**<br>(e.g. cow dung heals burns!)	
**43**	**Treatment**<br>(e.g. putting cow dung on a burn)	
**44**	**Effect**<br>(e.g. healing burns)	
**45**	**Basis**<br>(e.g. someone's personal experience)	
**46**	**Is the claim reliable?**<br>(e.g. no)	☐ Yes ☐ No ☐ Don't know/No response ☐ Not assessable

9. Table of categories on potential facilitators and barriers

**Teachers**		
**1**	Profiles and competences	Teacher's education and experience in relation to the lessons being taught
**2**	Understanding of the content being taught	Teachers' understanding of the context
**3**	Sufficient training	The extent to which the teachers received sufficient training in teaching the lessons
**4**	Self-efficacy	Teacher's confidence in teaching the lessons
**5**	Fit to the teacher's teaching style and context (e.g., class size)	Teachers' comfort or ability to adapt the instructions to their style and context
**6**	Attitudes	Teachers' attitude towards new resources (change), science, critical thinking and independent thinking by the student body (or their role as authorities in the classroom)
**7**	Beliefs	Teachers' beliefs about the methods or content (e.g., what treatments work or the concepts)
**8**	Emotions	Teachers' emotions, such as stress or anxiety
**9**	Motivation	Teachers' motivation to teach the material
**10**	Positive learning environment	Teachers' ability to create a positive learning environment; for example, encourage discussion, respond positively to questions, engage students
**Students**		
**11**	Literacy	Students' ability to read and understand the material
**12**	Attendance	Students' attendance or reasons for poor attendance (e.g., long distance to school or inability to pay school fees)
**13**	Motivation to learn	Students' motivation to learn the new material
**14**	Attitudes	Students' attitudes towards learning, towards authorities, towards science, towards critical thinking
**15**	Beliefs	Students' beliefs about the content (e.g., what treatments work or the concepts)
**16**	Home environment	The extent to which the student's home environment encourages or discourages learning from the lessons
**17**	Differentiated instruction	The extent to which students different learning needs are met
**18**	Peer influence	Positive or negative attitudes of other students towards the material
**Learning resources**		
**19**	Value of the material	The extent to which the materials are valued by the teachers and students
**20**	Compatibility with the curriculum	The extent to which the resources fits with the rest of the curriculum and how it is taught
**21**	Appropriateness of the material	The extent to which the resources are relevant, challenging and engaging
**22**	Credibility of the material	The extent to which the teachers and students perceive the resources as credible
**School system and environment**		
**23**	Time constraints	The extent to which there is sufficient time to accommodate introducing the new material
**24**	Competing priorities	The extent to which other priorities for the school, teachers or students limit introducing the resources (e.g., preparing for exams)
**25**	School organisation and management	The extent to which the school provides an environment that supports adoption of new subjects, resources and teaching methods
**26**	School resources, particularly human resources	The extent to which the school has adequate resources to introduce the new resources (e.g., human resources, student/teacher ratio, teacher workload, classroom space and classroom resources, such as blackboards and acoustics)
**27**	Attitudes and beliefs of head teacher and other teachers	Attitudes or beliefs of colleagues that influence the teacher's interest in and ability to teach the material
**28**	Parent and community involvement	Parents' attitudes towards the new resources or how things are done at the school
**29**	Regulations	Regulations (e.g., Ministry of Education policies and regulations) that affect introducing the new material
**30**	Political environment	Elements of the political environment that affect introducing the new material; for example, authoritarianism or teacher strikes
**31**	Bureaucracy	Bureaucratic arrangements that delay or limit introduction of the new materials, or facilitate introducing them
**32**	Incentives and disincentives	Incentives or disincentives to introduce the new resources for teachers or head teachers

S5.5 File. Guide for the semi-structured interviews with the students after a lesson

1. Objective

- Explore the students’ experience when using the IHC resources.

2. Instructions

1. Briefly introduce yourself.
2. Briefly introduce the IHC project:
   - “With this project, we want to help people make choices that matter to their health.”
3. Briefly explain what the interview is about:
   - “We are testing the project resources.”
   - “We are not testing you.”
   - “There are no right or wrong answers to any of the questions.”
   - “Your thoughts can help us improve the resources.”
   - “The interview will last approximately 30 minutes.”
   - “You can tell me any time if you do not wish to continue with the interview.”
4. Briefly explain that the interview’s audio will be recorded:
   - “We would like to record the interview to be able to review what we are going to discuss.”
   - “We will not use your name in the recording or notes.”
5. Make sure they have understood what the interview is about.
   - “Do you have any doubts or questions?”
6. Ask them if they wish to participate. Start audio recording if they approve.
7. Review the lesson's comic page by page and make questions as you go.

3. Questionnaire identification

1	**Date** (DD/MM/YYYY)	
2	**Researcher** (surname, name)	
3	**Student** (surname, name)	
4	**Teacher** (surname, name)	
5	**School**	
6	**Class**	
7	**Lesson number**	
8	**Interview start time**	
9	**Interview finish time**	
10	**Recording**	☐ Yes ☐ No ☐ Don't know/No response

4. Assessment of the students’ experience with the IHC resources

UNDERSTANDABILITY of the lesson

11	**How would you explain to a friend what John and Julie have LEARNED in this lesson?**	

**12**	**What is the HARDEST thing to understand in this lesson?**	
**13**	**Why?**	

DESIRABILITY in the lesson

**14**	**Do you think the lesson was INTERESTING?**	☐ Yes ☐ No ☐ Don't know/No response
**15**	**Why?**	
**16**	**What is the thing you LIKED THE MOST in this lesson?**	
**17**	**Why?**	
**18**	**What do you think was THE MOST BORING thing in this lesson?**	

19	**Why?**	

SUITABILITY of the lesson

20	**Do you think that what John and Julie have learnt in this lesson is APLICABLE to your day-to-day?**	☐ Yes ☐ No ☐ Don't know/No response
21	**Why?**	

USEFULNESS during the lesson

22	**Do you think <u>The Health Choices Book</u> is USEFUL to learn to think critically about health during the lesson?**	☐ Yes ☐ No ☐ Don't know/No response
23	**Do you think the <u>Activity Cards</u> are USEFUL to learn to think critically about health during the lesson? (if applicable)**	☐ Yes ☐ No ☐ Don't know/No response
24	**Do you think the <u>Checklist Poster</u> is USEFUL to learn to think critically about health during the lesson?**	☐ Yes ☐ No ☐ Don't know/No response
25	**Why?**	

5. Examples of a TREATMENT CLAIM

26	**Can you remember any TREATMENT CLAIM that you may have heard in your context (home, school, park)?**	☐ Yes ☐ No ☐ Don't know/No response

Please, describe the example.

27	**Claim**<br>(e.g. cow dung heals burns!)	
28	**Treatment**<br>(e.g. putting cow dung on a burn)	

29	**Effect**<br>(e.g. healing burns)	
30	**Basis**<br>(e.g. someone's personal experience)	
31	**Is the claim reliable?**<br>(e.g. no)	☐ Yes ☐ No ☐ Not sure

6. Suggestions to improve the lesson

32	**Would you change anything from this lesson?**	☐ Yes ☐ No ☐ Don't know/No response
33	**What would you change?**	
34	**Why would you change it?**	
35	**How would you change it?**	
