# Supplementary material for: Piloting the informed health choices resources in Barcelona primary schools: A mixed methods study
Source: PLoS One. 2023 Jul 7;18(7):e0288082. doi: 10.1371/journal.pone.0288082 (PMC10328314; doi:10.1371/journal.pone.0288082)
Supplement: S6 File — (PDF) [file pone.0288082.s006.pdf]

Piloting the Informed Health Choices resources in Barcelona primary schools: A mixed methods study

Supporting information

S6 File. Flow chart of schools' selection process

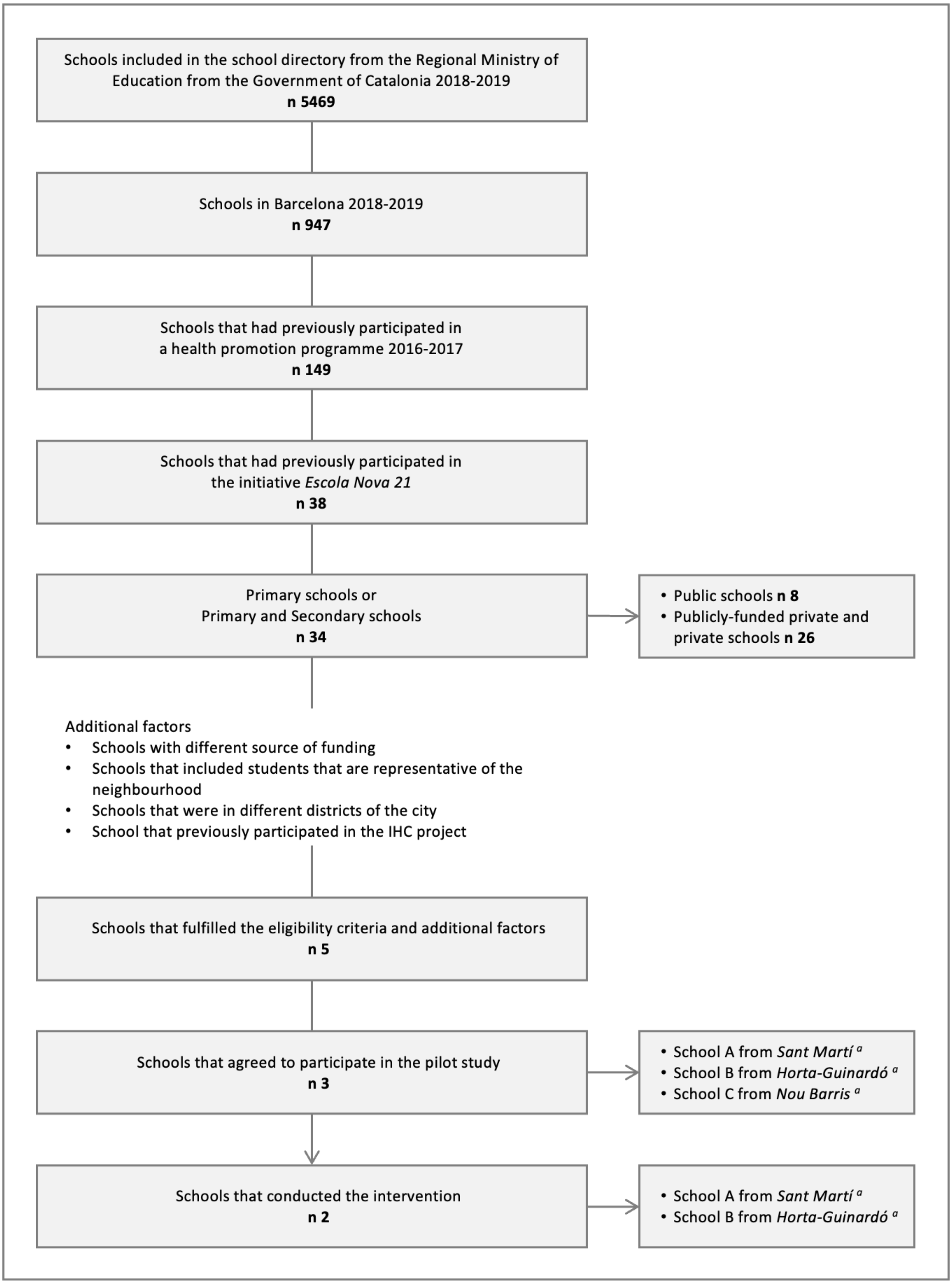

[a] *Sant Martí*, *Horta-Guinardó* and *Nou Barris* are districts of Barcelona (Spain).
