# Supplementary material for: Piloting the informed health choices resources in Barcelona primary schools: A mixed methods study
Source: PLoS One. 2023 Jul 7;18(7):e0288082. doi: 10.1371/journal.pone.0288082 (PMC10328314; doi:10.1371/journal.pone.0288082)
Supplement: S7 File — (PDF) [file pone.0288082.s007.pdf]

Piloting the Informed Health Choices resources in Barcelona primary schools: A mixed methods study

Supporting information

S7 File. Supplementary tables

S7.1 File. Intervention and data collection activities 2
S7.2 File. Results of the CLAIM test 3
S7.3 File. Technique used to teach the lessons 4
S7.4 File. Plan for teaching the lesson in School B during the second intervention 5
S7.5 File. Summarised results matrix 6
S7.6 File. Importance of the findings, implications for IHC resources, and recommendations 10

S7.1 File. Intervention and data collection activities

	1st intervention (2019-2020 school year)	2nd intervention (2020-2021 school year)	Overall
**Intervention, n**			
**Workshop**	1	N/A	**1**
**Lessons to the students**			
- Class A 5th grade School A	9	N/A	N/A
- Class B 5th grade School A	9	N/A	N/A
- Class A 5th grade School B [a]	2	N/A	N/A
- Class B 5th grade School B [a]	2	N/A	N/A
- Class A 4-5th grade School B [a]	N/A	5	N/A
- Class B 4-5th grade School B [a]	N/A	3	N/A
**Data collection activities, n**			
**Quantitative data collection**			
- Assessment of the IHC resources by the teachers before the lessons [b]	4	2	6
- Assessment of the lessons by the teachers after a lesson [b]	18	8	26
- Overall assessment of the IHC resources by the teachers at the end of the lessons [c]	2	N/A	N/A
**Qualitative data collection**			
- Non-participatory observations during the lessons	9	5	14
- Semi-structured interviews with the students after a lesson	9	5	14

Abbreviations: IHC: Informed Health Choices; N/A: Not applicable.

[a] School B adapted the lessons to include them in an extensive project-based learning (each lesson was divided into different sessions to work on other related additional contents).

[b] One teacher participated in the 1st and 2nd intervention.

[c] Teachers who completed all the lessons.

S7.2 File. Results of the CLAIM test

	1st intervention (2019-2020 school year) (n = 37) [a]	2nd intervention (2020-2021 school year) [b]	Overall
**Test score, n (%)**			
- Passing score [c]	36 (97.3)	N/A	N/A
- Mastery score [d]	23 (62.2)	N/A	N/A

Abbreviations: N/A Not applicable.

[a] Students who completed all the lessons.

[b] Students who did not complete all the lessons.

[c] Passing (a basic understanding of the concepts and how to apply them): 13 or more out of 24.

[d] Mastery (a clear understanding of the concepts and how to apply them): 20 or more out of 24.

S7.3 File. Technique used to teach the lessons

	1st intervention (2019-2020 school year)	2nd intervention (2020-2021 school year)	Overall
**Assessment of the lessons by the teachers after a lesson, n/N (%) [a]**			
- Step 1. Review the previous lesson [b, c]	15/16 (93.8)	5/6 (83.3)	20/22 (90.9)
- Step 2. Read the story (comic) [c, d]	14/14 (100)	6/8 (75)	20/22 (90.9)
- Step 3. Discuss the lesson [c]	18/18 (100)	8/8 (100)	26/26 (100)
- Step 4. Complete the activity [c]	17/18 (94.4)	5/8 (62.5)	22/26 (84.6)
- Step 5. Complete exercises [c]	16/18 (88.9)	8/8 (100)	24/26 (92.3)
- Other method and/or strategy to teach the lesson [c]	18/18 (100)	8/8 (100)	26/26 (100)

[a] n (observed step) / N (applicable lessons).

[b] Not applicable in lesson 1.

[c] School B adapted the lessons to include them in an extensive project-based learning (each lesson was divided into different sessions to work on other related additional contents).

[d] Not applicable in lessons 1 and 9.

S7.4 File. Plan for teaching the lesson in School B during the second intervention

Lesson	Activities	Group	Resources	Support
**Non-participatory observations during the lessons**				
**Lesson 1** [a]	Review last session	All students		
	Review textbook (illustrations)	All students	IHC Resources	Screen projector
	Exercise: Examples of treatments, positive and negative effects	Small group of students	Adaptation of the IHC resources	Personal notebook
	Closing remarks	All students		
**Lesson 2** [a]	Review last session	All students		
	Exercise: Worksheet with concepts and definitions	Individual student	Adaptation of the IHC resources	
	Read aloud the comic (animated version of Lesson 2)	All students	IHC Resources	Screen projector
	Exercise: Examples to identify personal experience	All students	Adaptation of the IHC resources	Blackboard
	Exercise: Worksheet to create a new comic character	Individual student	Adaptation of the IHC resources	
	Closing remarks	All students		
**Lesson 3** [a]	Presentation of the session	All students		
	Read aloud the comic	Small group of students	IHC Resources	Personal laptop
	Discussion about reading	Small group of students		
	Read aloud the comic	All students	IHC Resources	Screen projector
	Closing remarks	All students		
**Lesson 4** [a]	Presentation of the session	All students		
	Review last session	All students		
	Read aloud the comic	All students	IHC Resources	Screen projector
	Discussion about reading	All students		
	Exercise: Exercise to identify bad basis for claims	Small group of students	Adaptation of the IHC resources	Screen projector
	Closing remarks	All students		
**Lesson 5** [a]	Read aloud the comic	Small group of students	IHC Resources	Personal laptop
	Discussion about reading	Small group of students		
	Exercise: Reading comprehension worksheet	Small group of students	Adaptation of the IHC resources	Personal laptop

[a] School B adapted the lessons to include them in an extensive project-based learning (each lesson was divided into different sessions to work on other related additional contents).

S7.5 File. Summarised results matrix

	Quantitative findings	Qualitative findings	Integration findings
**Students' understandability**	**Assessment of the IHC resources by the teachers before the lessons**<br>Teachers expected that students would understand the content of the IHC resources (median score 4).<br>**Assessment of the lessons by the teachers after a lesson**<br>Students understood the content of the lessons (median score 5).<br>**Overall assessment of the IHC resources by the teachers at the end of the lessons**<br>Students understood the content of the IHC resources (median score 4.5).	**Non-participatory observations during the lessons**<br>Students understood, were interested, and were able to apply the content of the lessons. The textbook was useful for the students.<br><br>Relevant themes:<br>- Concept definition<br>- Other concepts<br>- Relationship between different concepts<br><br>**Semi-structured interviews with the students after a lesson**<br>All the students were able to explain what they had learned after the lessons. They enjoyed the lessons and thought that were interesting. They were able to apply the content of the lessons in their daily life.<br><br>Relevant themes:<br>- Lesson learning<br>- Concepts difficult to understand<br>- Interesting aspects of the lesson<br>- Boring aspects of the lesson<br>- Identification of treatments, and their advantages/disadvantages<br>- Suitability	**Convergence**<br>- Students' understandability<br>- Students' desirability<br>- Students' suitability<br>- Students' usefulness
**Students' desirability**	**Assessment of the IHC resources by the teachers before the lessons** Teachers expected that students would be slightly less interested in the content of the IHC resources (median score 3.5).<br>**Assessment of the lessons by the teachers after a lesson**<br>Students were interested in the content of the lessons (median score 4).<br>**Overall assessment of the IHC resources by the teachers at the end of the lessons**<br>Students were interested in the content of the IHC resources (median score 4.5).		
**Students' suitability**	**Assessment of the IHC resources by the teachers before the lessons**<br>Teachers expected that students would apply the content of the IHC resources to their daily life (median score 4).<br>**Assessment of the lessons by the teachers after a lesson**<br>Students were able to apply the content of the lessons to their daily life (median score 4).<br>**Overall assessment of the IHC resources by the teachers at the end of the lessons**<br>Students were able to apply the content of the IHC resources to their daily life (median score 4.5).		
**Students' usefulness**	**Assessment of the IHC resources by the teachers before the lessons**<br>Teachers expected that IHC resources would be useful for students (median score 4).<br>**Assessment of the lessons by the teachers after a lesson**<br>Teachers reported that textbook was useful for students (median score 5), despite there was variability (range 1-5). Teachers reported that activity cards were useful for students in lesson 7 (median score 5). Teachers reported that poster wast not useful for students (median score 1).<br>**Overall assessment of the IHC resources by the teachers at the end of the lessons**<br>Teachers reported that the IHC resources were useful for students (median score 5).		

	Quantitative findings	Qualitative findings	Integration findings
**Teachers' understandability**	**Assessment of the IHC resources by the teachers before the lessons**<br>Teachers understood the content of the IHC resources (median score 5).<br>**Assessment of the lessons by the teachers after a lesson**<br>Teachers understood the content of the lessons (median score 5).<br>**Overall assessment of the IHC resources by the teachers at the end of the lessons**<br>Teachers understood the content of the IHC resources (median score 5).	**Non-participatory observations during the lessons**<br>Teachers understood, were interested, and were able to apply the content of the lessons. They rarely used their printed copy of the textbook or of the teachers' guide during the lessons.<br><br>Relevant themes:<br>- Concept definition<br>- Classroom dynamics<br>- Maturity of discussion with lesson progression	**Convergence**<br>- Teachers' understandability<br>- Teachers' desirability<br>- Teachers' suitability<br><br>**Discrepancy**<br>We observed discordance in qualitative and quantitative findings about the usefulness of the IHC resources for teachers. Before starting the lessons, they expected the textbook to be useful (median score 4.5); after the lessons and at the end of the lessons, they reported that the textbook and the teachers' guide were useful (median score 5). However, during the NPOs we observed that they rarely used their printed copy of the textbook or the teachers' guide. This discrepancy could be due to the teachers rating the IHC resources as highly useful because they used them to prepare the lessons (adaptation of the IHC resources); instead, observers rated the IHC resources as less useful because teachers made many adaptations to them.
**Teachers' desirability**	**Assessment of the IHC resources by the teachers before the lessons**<br>Teachers were interested in the content of the lessons (median score 4.5).<br>**Assessment of the lessons by the teachers after a lesson**<br>Teachers were interested in the content of the lessons (median score 5).<br>**Overall assessment of the IHC resources by the teachers at the end of the lessons**<br>Teachers were interested in the content of the IHC resources (median score 5).		
**Teachers' suitability**	**Assessment of the IHC resources by the teachers before the lessons**<br>Teachers were able to apply the content of the IHC resources to their daily life (median score 4).<br>**Assessment of the lessons by the teachers after a lesson**<br>Teachers were able to apply the content of the lessons to their daily life (median score 5).<br>**Overall assessment of the IHC resources by the teachers at the end of the lessons**<br>Teachers were able to apply the content of the IHC resources to their daily life (median score 4.5).		
**Teachers' usefulness**	**Assessment of the IHC resources by the teachers before the lessons**<br>Teachers expected that the textbook to be the most useful resource (median score 4.5) compared to the teachers' guide, the activity cards, or the poster (median score 3.5, 3.5, and 3 respectively).<br>**Assessment of the lessons by the teachers after a lesson**<br>Teachers reported that all the IHC resources were useful (median score 5), except the poster (median score 1). despite there was variability Although there was great variability in the usefulness (range 1-5).<br>**Overall assessment of the IHC resources by the teachers at the end of the lessons**<br>Teachers reported that the textbook and the teachers' guide were the most useful resources (median score 5).		

	Quantitative findings	Qualitative findings	Integration findings
**Technique used to teach the lessons**	**Assessment of the lessons by the teachers after a lesson**<br>Teachers used the techniques of the plan proposed in the IHC resources to teach the lessons: review of the previous lesson, reading the lesson's comic, a discussion, the completion of the activity/exercises.	**Non-participatory observations during the lessons**<br>Teachers from School A almost always followed the plan proposed in the IHC resources, which included a review of the previous lesson, reading the lesson's comic, a discussion, and the completion of the activity/exercises.<br><br>Teachers from the School B implemented the lessons as project-based learning, and they substantially changed the plan proposed in the IHC resources. Nevertheless, they maintained some of the steps of the plan, such as reading the lesson's comic, the discussion, and completion of exercises.<br><br>In both schools, teachers used other methods or strategies to teach the lessons.<br><br>Relevant themes<br>- Adaptation of the IHC resources<br>- Use of ICT tools<br><br>**Assessment of the lessons by the teachers after a lesson**<br>All teachers used other methods or strategies to teach the lessons.<br><br>Relevant themes<br>- Adaptation of the IHC resources<br>- Use of ICT tools	**Complementarity**<br>We observed that qualitative findings complemented the quantitative findings about how teachers delivered the lessons. Teachers used the techniques of the proposed plan in the IHC resources (review of the previous lesson, reading the lesson's comic, a discussion, and the completion of the activity/exercises), and other methods or strategies. During the NPOs, we observed differences in the implementation of the techniques of the proposed plan in the IHC resources between participating schools, and we captured in detail how other strategies and methods were implemented.
**Facilitators to teach the lessons**	**Assessment of the lessons by the teachers after a lesson**<br>Facilitators n=274<br>The most frequent identified facilitators:<br>- Teachers: fit to the teacher's teaching style and context, self-efficacy, understanding of the content being taught<br>- Students: attitudes, beliefs, motivation to learn<br>- IHC resources: credibility of the material	**Non-participatory observations during the lessons**<br>Teachers were deeply engaged to teach the lessons (attitudes, fit to the teacher's teaching style and context, self-efficacy, motivation, and positive learning environment).<br><br>Students also contributed to the adequate progress of the lessons (literacy, motivation to learn, peer influence, and attitudes).	**Convergence**<br>- Facilitators to teach the lessons<br>- Barriers to teach the lessons
**Barriers to teach the lessons**	**Assessment of the lessons by the teachers after a lesson**<br>Barriers n=29<br>The most frequent identified barriers:<br>- Students: peer influence, differentiated instruction<br>- School system and environment: time constraints	**Non-participatory observations during the lessons**<br>We identified few barriers, some of these were related to the students (peer influence and attitudes) and the school system and environment (school organisation and management).<br><br>We observed circumstances that were both, a facilitator and a barrier.	

	Quantitative findings	Qualitative findings	Integration findings
**Examples of claims about treatment effects**		During the first intervention, students and teachers identified examples from their daily lives, mainly based on personal experiences and non-serious illnesses.<br>During the second intervention, students and teachers gave several examples of treatment claims related to COVID-19.	
**Suggestions to improve the lessons**		**Assessment of the lessons by the teachers after a lesson**<br>**Overall assessment of the IHC resources by the teachers at the end of the lessons**<br>Teachers suggested ideas to improve the lessons based on the activities that they implemented to teach the lessons or their teaching experience.<br><br>Relevant themes:<br>- Adaptation of the IHC resources to promote students' participation<br>- Use of ICT tools<br>- Modifications or combination of some of the lessons<br>- Comic animation<br><br>**Semi-structured interviews with the students after a lesson**<br>Some of the students made suggestions to improve the lessons and highlighted that the comic was what interested them the most.<br><br>Relevant themes:<br>- Interest in the comic	

Abbreviations: IHC: Informed Health Choices; ICT: Information and Communication Technologies.

S7.6 File. Importance of the findings, implications for IHC resources, and recommendations

	Findings	Importance of the findings	Recommendations
1	One school (School A) completed all the lessons during the first intervention (January-March 2020).<br><br>The other school (School B) only completed two lessons during the first intervention (February-March 2020), and 3-5 lessons during the second intervention (April-June 2021)	Very important negative finding	It is important that teachers plan an accurate timetable to allow enough time to complete all lessons.
2	During the lessons, students understood, were interested, and were able to apply the content of the lessons.<br><br>The textbook was useful for the students.<br><br>Some of the interviewed students also made suggestions to improve the lessons, all of them to highlight what was of most interest to them in the lessons: the comic included in the textbook.	Positive finding	It is important that teachers use the comic of the textbook to engage students' interest.
3	The participating teachers used different techniques to teach the lessons.<br><br>In both schools, teachers used other methods or strategies to teach the lessons.	Very important positive finding	Teachers can adapt the IHC resources to the teaching strategy and educational project of each school.
4	All teachers developed other activities/exercises and work materials based on the IHC resources to promote students' participation.	Very important negative finding	The IHC resources need to include activities that promote student participation.<br><br>It is important to collect, in an open database, examples, activities and materials developed based on the IHC resources and elaborated in different settings; in order to share teaching and learning experiences and avoid duplication of efforts.
5	Teachers and students used ICT tools to support teaching of the lessons.	Very important constructive finding	The availability of ICT tools in the classroom can support the development of more participatory and interactive IHC resources.<br><br>The availability of ICT tools in the classroom can replace the use of paper based IHC resources.

Abbreviations: IHC: Informed Health Choices; ICT: Information and Communication Technologies.

Coding of the importance of the findings [1]

Code	Description
Very important negative finding	A problem that we should address for the resources to be effective
Important negative finding	A problem that we should probably address for part of the resources to be effective
Negative finding	A problem that we can easily address and probably will not prevent the resources from being effective
Very important positive finding	Praise that probably should inspire changes
Important positive finding	Praise that maybe should inspire changes
Positive finding	Praise that probably should not inspire changes
Very important constructive finding	A suggestion that probably should inspire changes
Important constructive finding	A suggestion that maybe should inspire changes
Constructive finding	A suggestion that probably should not inspire changes

Reference: Nsangi A, Semakula D, Rosenbaum SE, Oxman AD, Oxman M, Morelli A, et al. Development of the informed health choices resources in four countries to teach primary school children to assess claims about treatment effects: a qualitative study employing a user-centred approach. Pilot Feasibility Stud. 2020;6:18.
