# Supplementary material for: Piloting the informed health choices resources in Barcelona primary schools: A mixed methods study
Source: PLoS One. 2023 Jul 7;18(7):e0288082. doi: 10.1371/journal.pone.0288082 (PMC10328314; doi:10.1371/journal.pone.0288082)
Supplement: S8 File — The S8 File is available at: https://doi.org/10.6084/m9.figshare.23148563.v1. (PDF) [file pone.0288082.s008.pdf]

Piloting the Informed Health Choices resources in Barcelona primary schools: A mixed methods study

Supporting information

May 2023

Samsó Jofra L, Alonso-Coello P, Cánovas Martínez E, de Britos Marsal C, Gallego Iborra A, Niño de Guzman Quispe EP, *et al*. Piloting the Informed Health Choices resources in Barcelona primary schools: A mixed methods study - Supporting information. 2023.
Corresponding author: lsamso@santpau.cat

S8 File. Activities and work materials developed by teachers

S8.1 File. Worksheet with concepts and definitions (only available in Spanish)....2
S8.2 File. Cards with the treatment illustrations from the textbook....3
S8.3 File. Diagram with treatment advantages and disadvantages (only available in Spanish)....4
S8.4 File. Worksheet with examples about claims....5
S8.5 File. Worksheet with concepts and definitions....6
S8.6 File. Exercise to identify bad basis for claims....7
S8.7 File. Worksheet with exercises....10
S8.8 File. Health survey....13
S8.9 File. Questionnaire about comic....15
S8.10 File. Worksheet to create a new comic character....16
S8.11 File. English vocabulary exercise about common symptoms....17
S8.12 File. Worksheet to develop a health word search....18
S8.13 File. Reading comprehension worksheet....19
S8.14 File. Exercise to develop a dialogue between a doctor and a patient....21
S8.15 File. Exercise to listen dialogues between doctors and patients....22

S8.1 File. Worksheet with concepts and definitions (only available in Spanish)

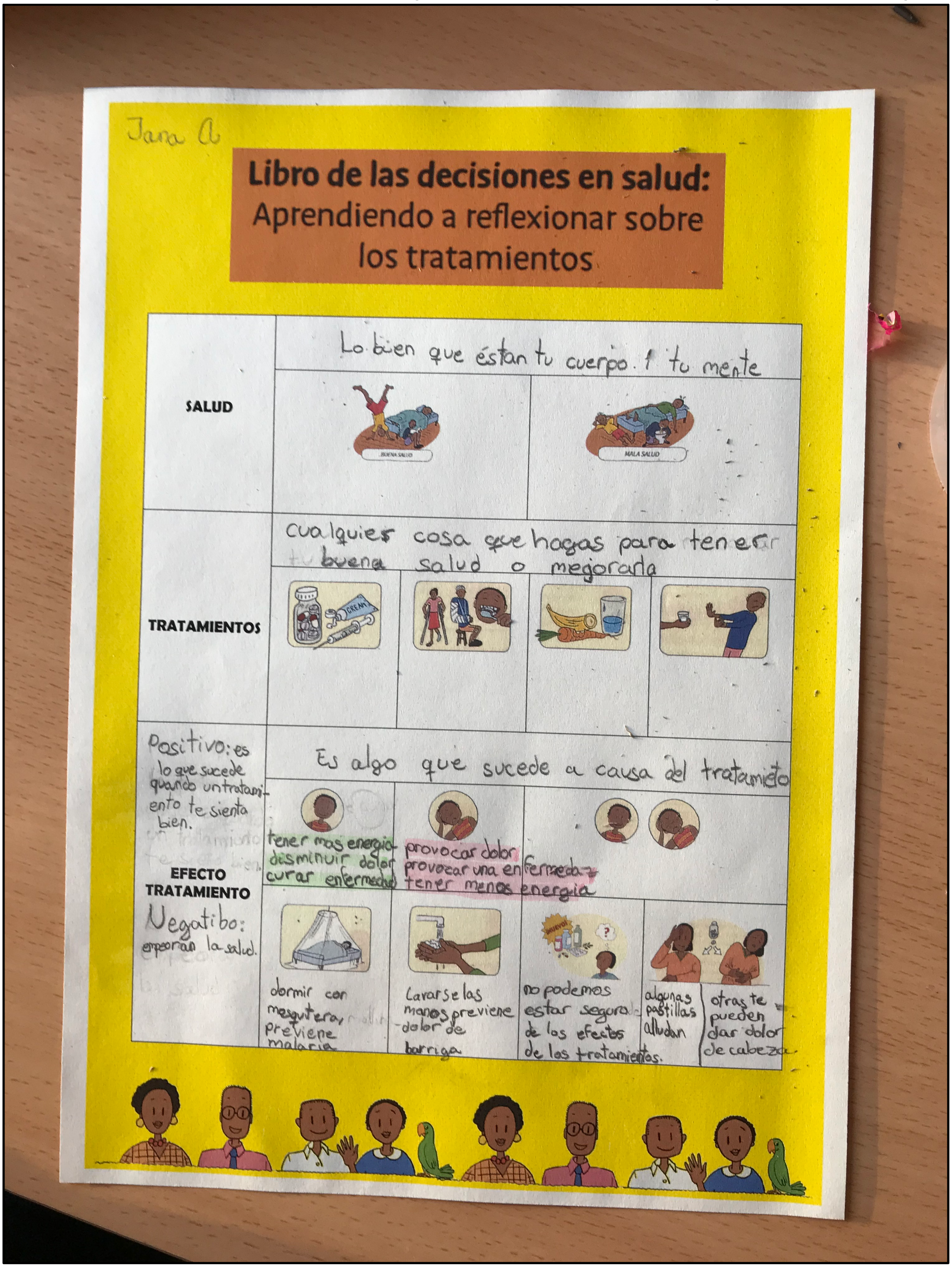

S8.2 File. Cards with the treatment illustrations from the textbook

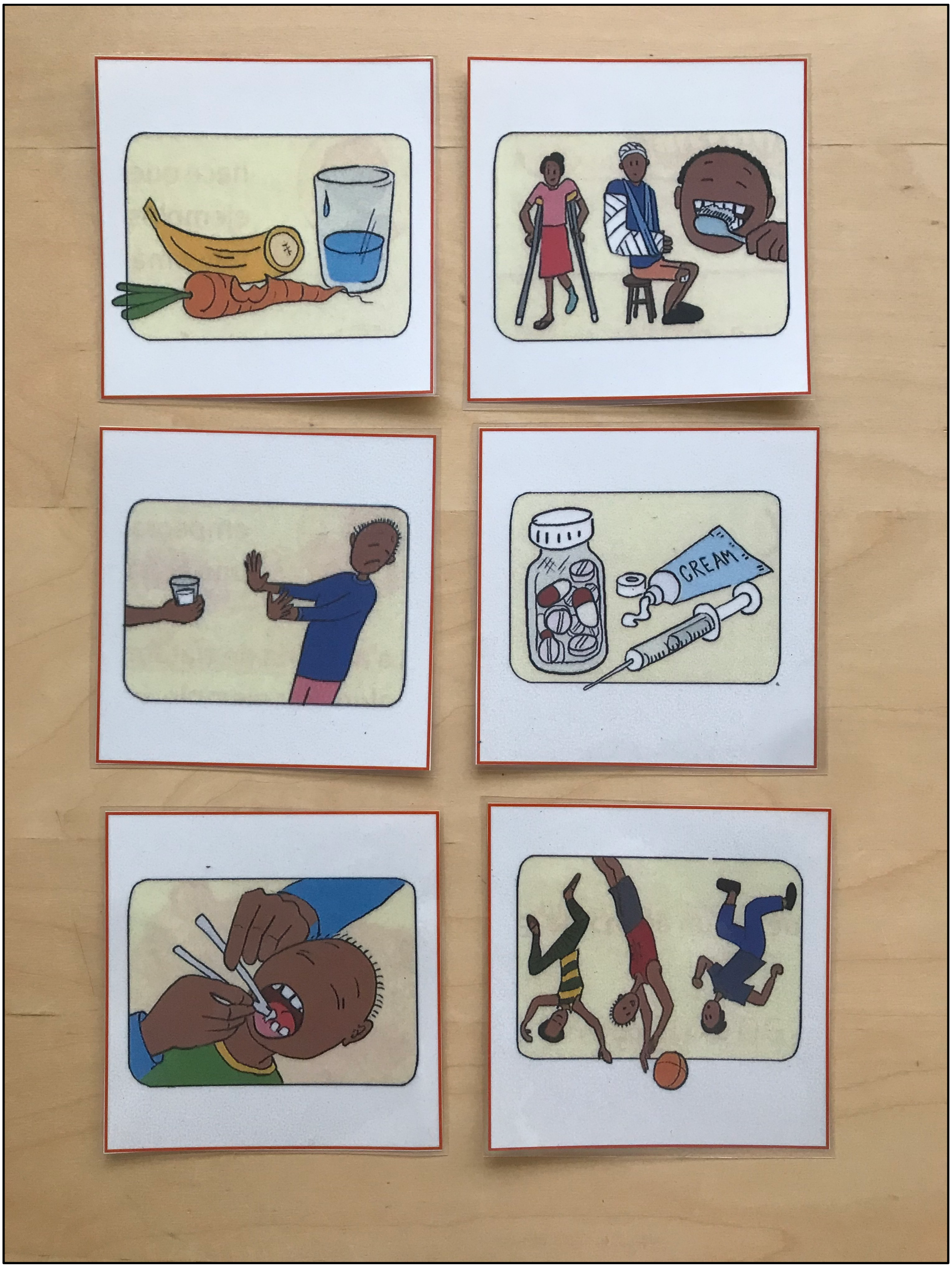

S8.3 File. Diagram with treatment advantages and disadvantages (only available in Spanish)

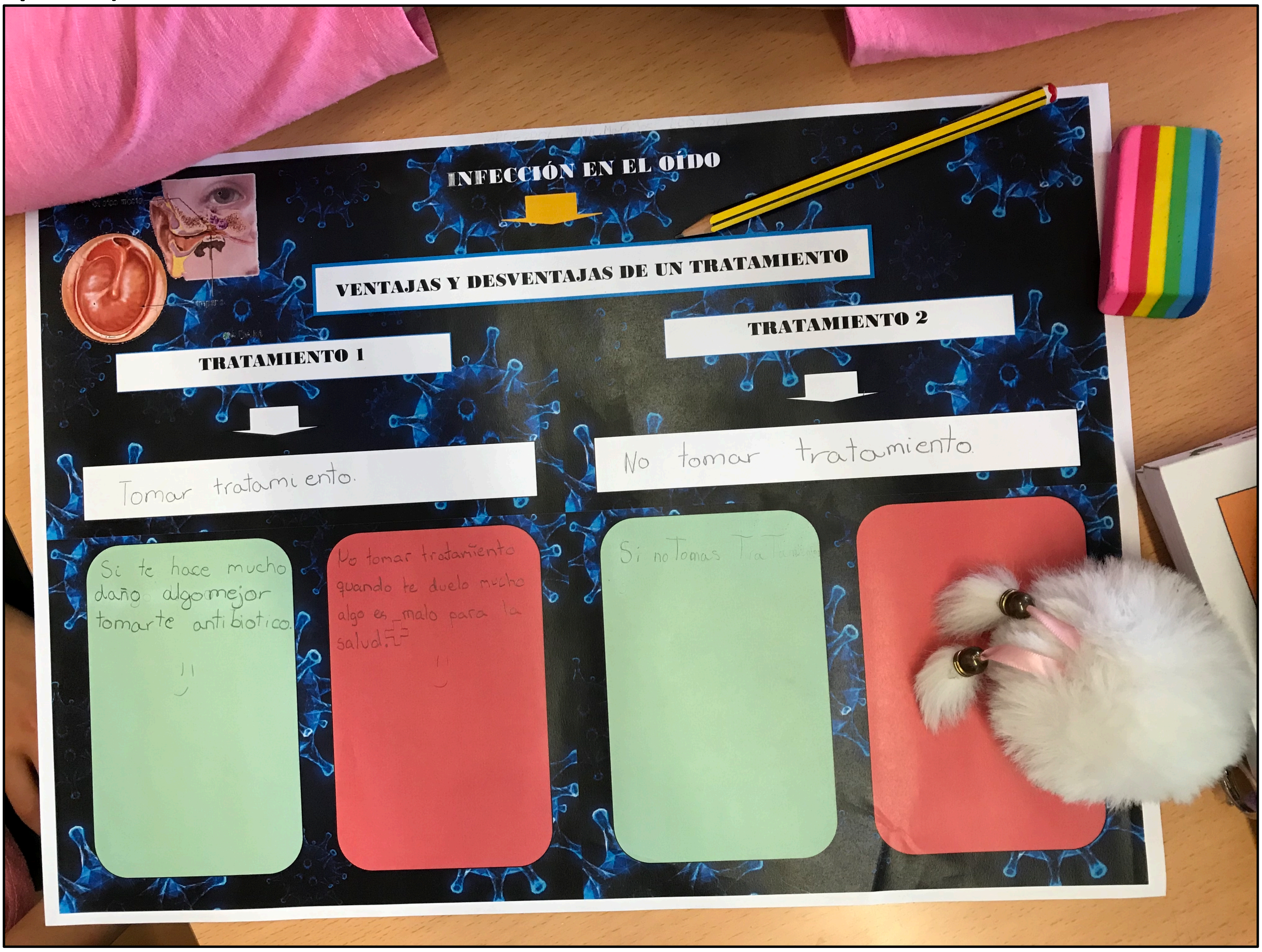

S8.4 File. Worksheet with examples about claims

CLAIMS

- If a jellyfish bites you, you can put pee on your skin or your bite.
- If you get burn, you can put Aloe Vera
- If you have a stomach ache, you can drink CocaCola.
- If you have a headache, you can drink some water.
- If you have a cough, you can take Dalsy.
- If you have something in your eye, you can put drops on it.
- If you get burned, you can add salt.
- If you have pain in your bones, you can take Dalsy.
- If you get drunk, you can take a coffee cup with salt.
- If a wound is itching, that means it is being cured.
- If you hit something, you need to put ice.

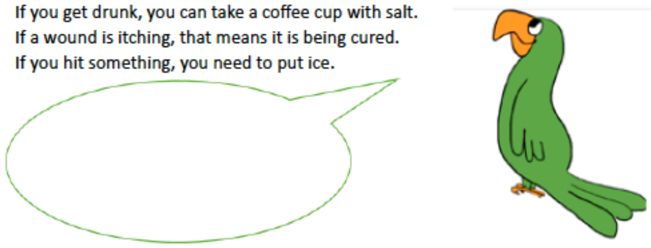

S8.5 File. Worksheet with concepts and definitions

KEY WORDS LESSON 2

Activity: Match the words with their meaning.

WORDS	MEANINGS
CLAIM	A claim with a bad basis.
PERSONAL EXPERIENCE using a treatment	Something that someone says that can be right or wrong.
BASIS for a claim	Something that happened to someone using a treatment.
UNRELIABLE claim	Support foundation or reason for the claim.

S8.6 File. Exercise to identify bad basis for claims

Activity: BAD BASIS for CLAIMS

MANY PEOPLE SAY SO	PERSONAL EXPERIENCE	HOW NEW A TREATMENT IS	COST OF THE TREATMENT	SOMEONE WHO SELLS A TREATMENT SAYS SO

CLAIM 1

Sarah put cow dung on a burn once and the burn went away. Now Sara says that cow dung heals burns.

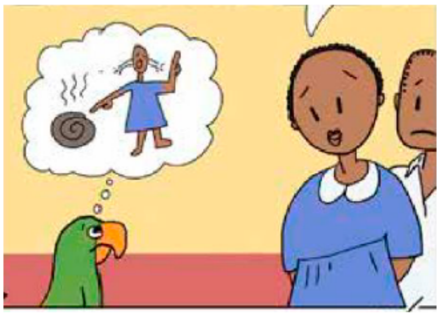

MANY PEOPLE SAY SO	PERSONAL EXPERIENCE	HOW NEW A TREATMENT IS	COST OF THE TREATMENT	SOMEONE WHO SELLS A TREATMENT SAYS SO

CLAIM 2

There are three different types of bottles of water at the shop. Clare buys the most expensive one because she says that it is better for your health if it costs more money.

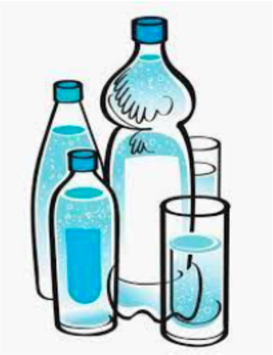

MANY PEOPLE SAY SO	PERSONAL EXPERIENCE	HOW NEW A TREATMENT IS	COST OF THE TREATMENT	SOMEONE WHO SELLS A TREATMENT SAYS SO

CLAIM 3

Mark wears new trainers to do Physical Education at school. He wins a race and he says that he runs faster because he is wearing new shoes.

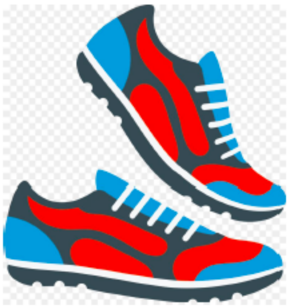

MANY PEOPLE SAY SO	PERSONAL EXPERIENCE	HOW NEW A TREATMENT IS	COST OF THE TREATMENT	SOMEONE WHO SELLS A TREATMENT SAYS SO

CLAIM 4

Alice eats potatoes everyday. She says it makes her stronger because many people told her so.

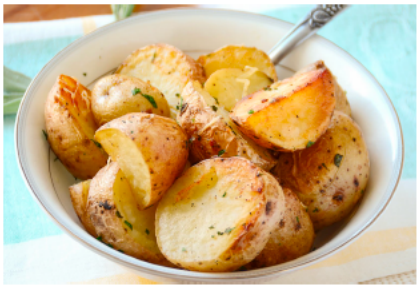

MANY PEOPLE SAY SO	PERSONAL EXPERIENCE	HOW NEW A TREATMENT IS	COST OF THE TREATMENT	SOMEONE WHO SELLS A TREATMENT SAYS SO

CLAIM 5

Christopher buys some candles. He says that if you use the candles, mosquitoes won't bite you. He says it because a shop assistant said so.

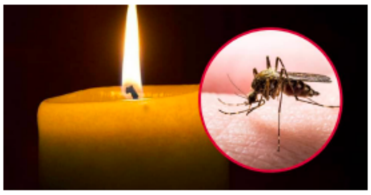

MANY PEOPLE SAY SO	PERSONAL EXPERIENCE	HOW NEW A TREATMENT IS	COST OF THE TREATMENT	SOMEONE WHO SELLS A TREATMENT SAYS SO

S8.7 File. Worksheet with exercises

Activity Lesson 5

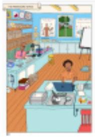

1) Look at the PROFESSORS' OFFICE picture and write True or False.

	True or False?
a. John and Julie are at the University.	
b. Professor Fair is using the computer.	
c. Professor Compare is writing on a sheet of paper.	
d. There is a skeleton at the professors' office.	

2) Look for these words in **Kiswahili** and write the translation in **English**.

**KISWAHILI**	**ENGLISH**
Kupima	
Kulinganisha	
Swali la utafiti	

3) Write **numbers 1, 2 and 3** to order the steps that health researchers do.

NUMBER	STEPS
	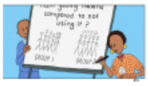 Make one GROUP OF PEOPLE for each treatment.
	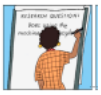 Write a QUESTION of a claim.
	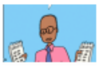 MEASURE what happened.

4) Each face like this is a person with **malaria**: ☹

Measure the difference between the groups. Here is one **example**.

EXAMPLE:

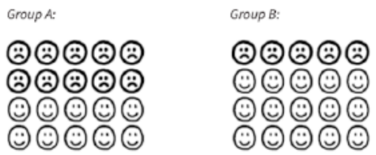

How many people had malaria in each group?

Group A: *10* out of 20.

Group B: *5* out of 20

What was the difference between the groups?

There were *5* more people with malaria out of 20 in Group *A*.

EXERCISE 1

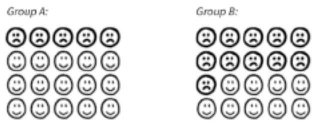

How many people had malaria in each group?

Group A: ____ out of 20.

Group B: ____ out of 20

What was the difference between the groups?

There were _____more people with malaria out of 20 in Group ____.

EXERCISE 2

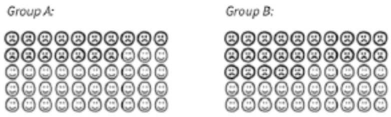

How many people had malaria in each group?

Group A: ___ out of 50.

Group B: ___ out of 50

What was the difference between the groups?

There were _____more people with malaria out 50 in Group ___.

EXTRA! Complete these sentences with words from the box.

QUESTION	MALARIA	RESEARCHERS	MEASURE	GROUP OF PEOPLE

a) Health __________ study treatments by comparing them.
b) Namuli's claim was about a machine to stop you to get __________.
c) First, health researchers take a claim and turn it into a _________.
d) After the question, they make one _______ of people for each treatment.
e) Finally, they have to ________ what happened.

S8.8 File. Health survey

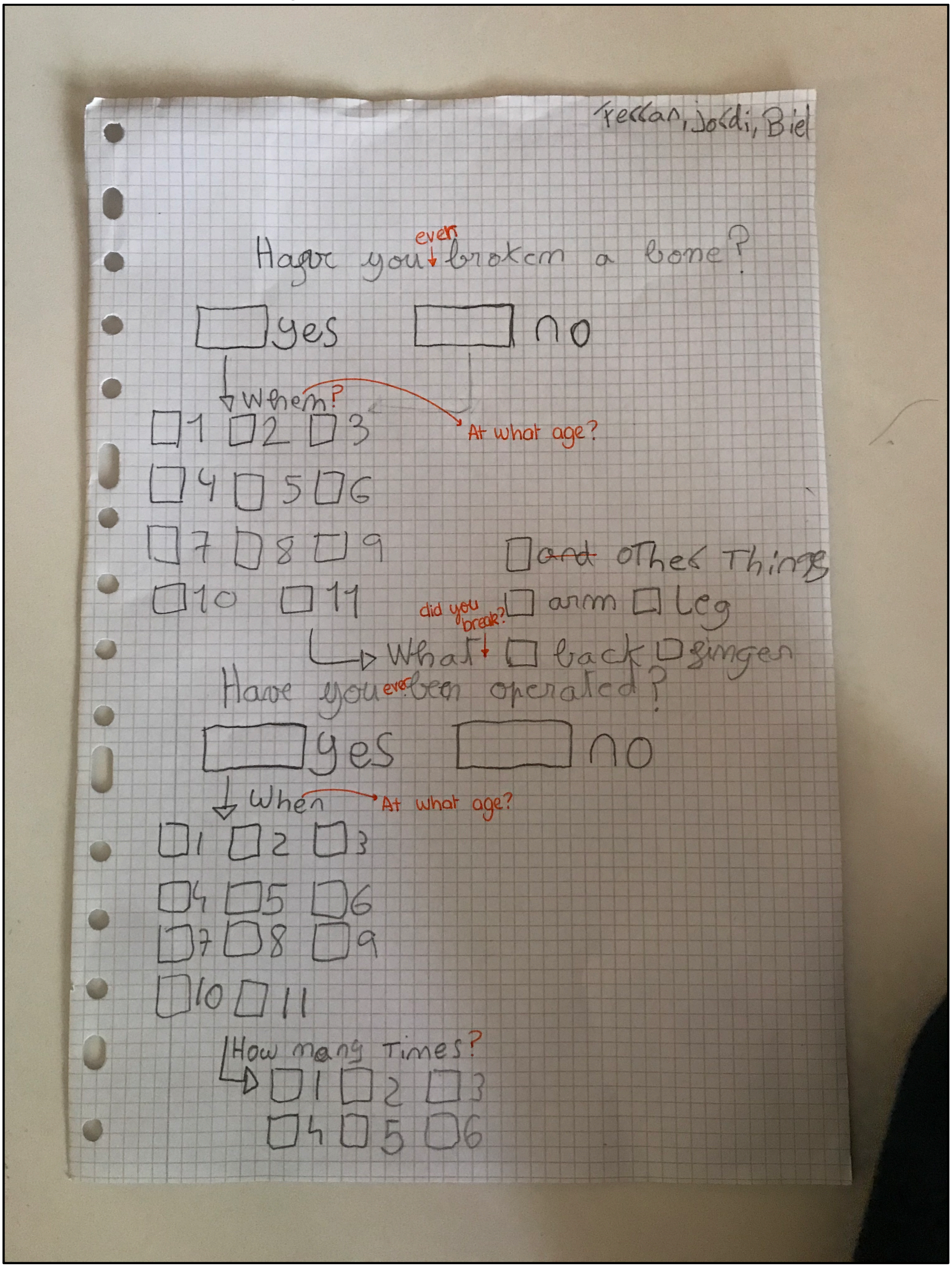

Ferran, Jordi, Biel

Have you ever broken a bone?

☐ yes ☐ no

When? → At what age?

☐ 1 ☐ 2 ☐ 3

☐ 4 ☐ 5 ☐ 6

☐ 7 ☐ 8 ☐ 9

☐ 10 ☐ 11

What did you break? ☐ arm ☐ leg ☐ back ☐ ginger ☐ ~~and~~ other Things

Have you ever been operated?

☐ yes ☐ no

When → At what age?

☐ 1 ☐ 2 ☐ 3

☐ 4 ☐ 5 ☐ 6

☐ 7 ☐ 8 ☐ 9

☐ 10 ☐ 11

How many Times?

☐ 1 ☐ 2 ☐ 3

☐ 4 ☐ 5 ☐ 6

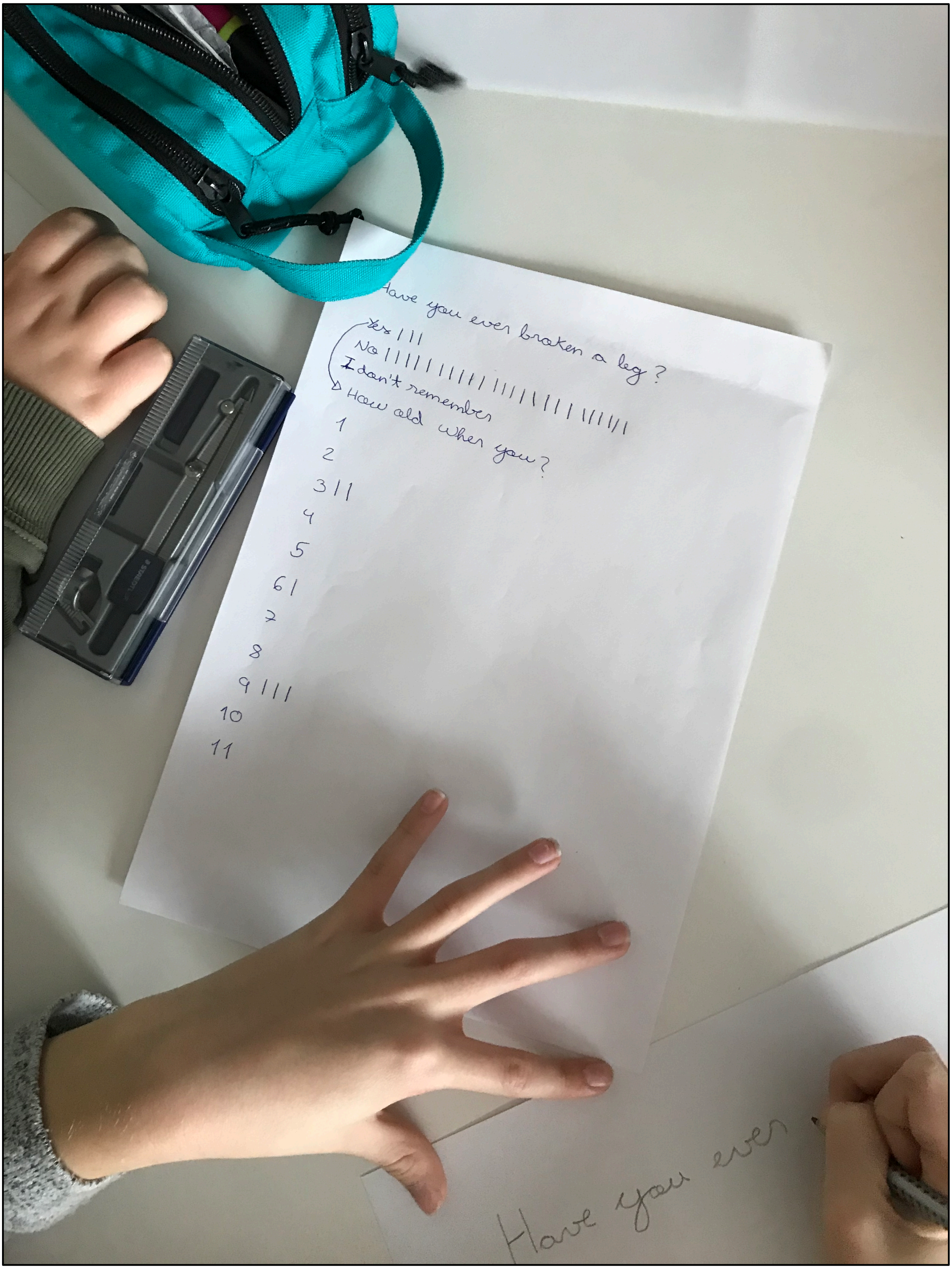
ave you ever broken a leg?
Yes |||
No
I don't remember
How old when you?
1
2
3 ||
4
5
6 |
7
8
9 |||
10
11
Have you ever

S8.9 File. Questionnaire about comic

COMIC: PEER-ASSESSMENT

		YES/NO
Does the comic include …?	thought bubbles	
	speech bubbles	
	narrator	
Does the comic show…?	a well explained treatment	
	a good effect	
	a bad effect	
Is the comic…?	her/his best work	
	done in pencil	
	a good explanation of treatment & effects	
	original	

S8.10 File. Worksheet to create a new comic character

CASTING: A new character

INSTRUCTIONS: The comic editors are looking for new characters for this comic! Invent a new character for the comic that we are reading. It can be John's friend, a teacher from school, some family member, a health researcher, an animal, someone from the market… Complete the information about the character and then write a short description.
***Optional:** Add a picture of the new character made by you.

NAME:
AGE:
JOB:
COUNTRY: (Uganda)
PHYSICAL DESCRIPTION:
HOBBIES:
PERSONALITY:
**WHY IS IT A GOOD CHARACTER FOR THE COMIC?**

My name is ________ and I am _______ years old. I am a ___________ and I am from ___________.

I am ____.
My hobbies are ____.

I am ____.

I am a good character for the comic because ____________________________________
____.

S8.11 File. English vocabulary exercise about common symptoms

escola virolai

What's the matter?

1. Write the words. Then add more body words.

arm shoulder stomach leg foot
head teeth neck back hand

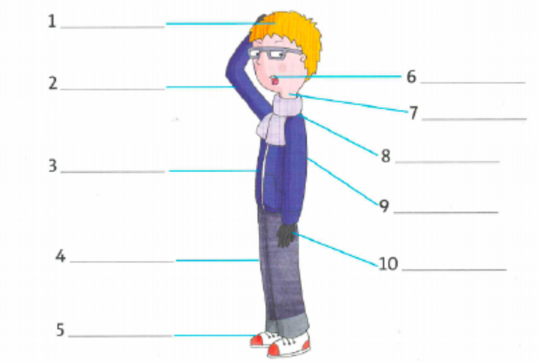

2. What are they saying? Circle.

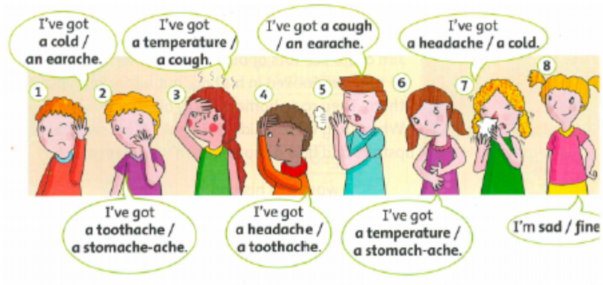

S8.12 File. Worksheet to develop a health word search

Creator: Player:

HEALTH WORD SEARCH

Instructions: Choose 10 words related to HEALTH and create a word search with them!

The 10 words are...

Now look for them!

S8.13 File. Reading comprehension worksheet

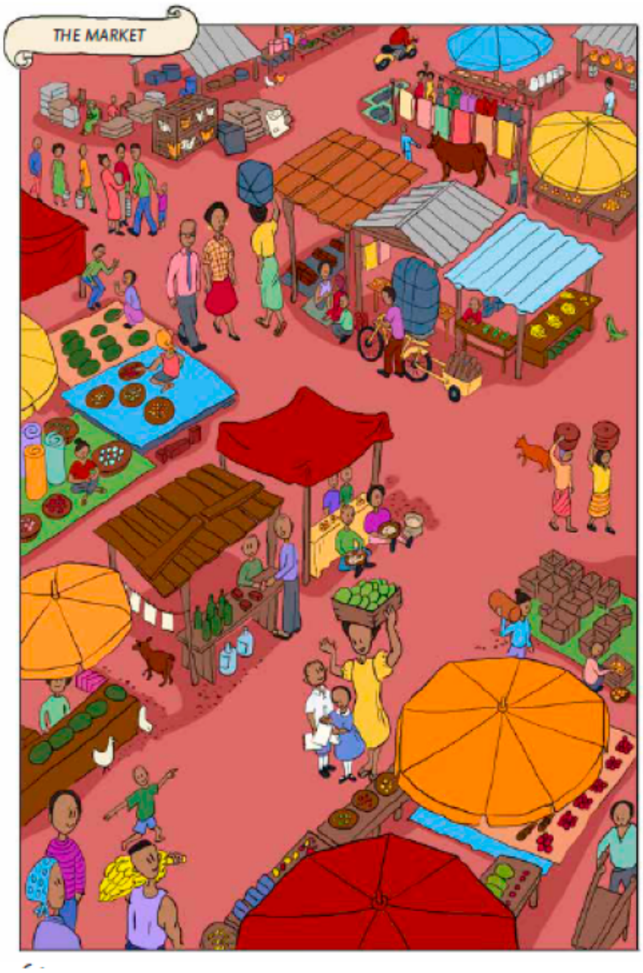

AT THE MARKET

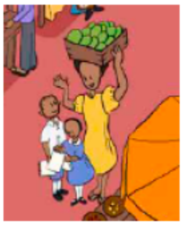

1) **True or false?**
   a) A boy is carrying some bananas. _____
   b) Julie and John are buying oranges. ______
   c) Kasuku is flying. _____
   d) There is a motorbike. _____
   e) Professor Francis is wearing glasses. ______

2) **Answer these questions about the Market picture.**
   a) How many watermelons are there?
   b) How many people are there?
   c) How many chickens are there?
   d) How many bikes can you see?

3) **Complete the sentences with the correct word from the box.**

listening	walking	pointing	looking	buying

a) Professors Fair and Compare are ______________ in the market.
b) Kasuku is ___________ at the bananas.
c) Julie and John are ___________ to a woman.
d) The boy with a green t-shirt is __________ to John and Julie.
e) There is a boy _______ a watermelon.

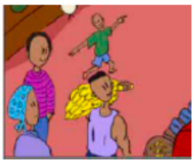

S8.14 File. Exercise to develop a dialogue between a doctor and a patient

DIALOGUE

INSTRUCTIONS: Write a dialogue between a DOCTOR and a PATIENT. Colour in **RED** the sicknesses and in **GREEN** the **treatment** that the doctor recommends. You have to write a minimum of 10 sentences in total. Choose also a TITLE for the dialogue and add a PICTURE about the dialogue at the end.

TITLE:

S8.15 File. Exercise to listen dialogues between doctors and patients

LISTENING DIALOGUES

Instructions: Click on this link AUDIOS DIALOGUES and listen to a minimum of 6 audios of other classmates. Then complete the table with the information of the dialogues.

AUTHORS	HEALTH PROBLEM	TREATMENT
